# Supplementary material for: Co-aggregation with Apolipoprotein E modulates the function of Amyloid-β in Alzheimer’s disease
Source: Nat Commun. 2024 Jun 1;15:4695. doi: 10.1038/s41467-024-49028-z (PMC11144216; doi:10.1038/s41467-024-49028-z)
Supplement: Supplementary file 1 — Supplementary Information [file 41467_2024_49028_MOESM1_ESM.pdf]

Supplementary information

Co-aggregation with Apolipoprotein E modulates the function of Amyloid-β in Alzheimer's disease

Zengjie Xia[1,2,12], Emily E. Prescott[3,12], Agnieszka Urbanek[3,12], Hollie E, Wareing[3], Marianne C King[3], Anna Olerinyova[3], Helen Dakin[1,2,4], Tom Leah[3], Katy A. Barnes[3], Martyna M Matuszyk[3], Eleni Dimou[1,2], Eric Zuo[1,2], Yu P. Zhang[1,2], Jeff Y.L. Lam[1,2], John S. H. Danial[1,2,5], Michael R. Strickland[6,7], Hong Jiang[6], Peter Thornton[8], Damian C. Crowther[8], Sohvi Ohtonen[-9], Mireia Gómez-Budia[9], Simon M. Bell[3,10], Laura Ferraiuolo[3], Heather Mortiboys[3,10,11], Adrian Higginbottom[3,10], Stephen B. Wharton[3,10], David M. Holtzman[6], Tarja Malm[9], Rohan T. Ranasinghe[1,2,*], David Klenerman[1,2,*], Suman De[3,10,11*]

[1]Yusuf Hamied Department of Chemistry, University of Cambridge, Cambridge, UK

[2]UK Dementia Research Institute at University of Cambridge, Cambridge, UK

[3]Sheffield Institute for Translational Neuroscience, Division of Neurosciences, University of Sheffield, Sheffield, S10 2HQ, UK

[4]Clinical Neurosciences, University of Cambridge, Cambridge CB2 0QQ, UK.

[5]SUPA School of Physics and Astronomy, University of St Andrews, North Haugh, St Andrews, KY16 9SS

[6]Department of Neurology, Hope Center for Neurological Disorders, Knight ADRC, Washington University School of Medicine, St. Louis, MO, USA

[7]Department of Pathology and Immunology, Washington University School of Medicine, St. Louis, MO, USA

[8]Neuroscience, BioPharmaceuticals R&D, AstraZeneca, Cambridge, UK.

[9]A.I. Virtanen Institute for Molecular Sciences, University of Eastern Finland, Kuopio, Finland

[10]Neuroscience Institute, University of Sheffield, Sheffield, S10 2TN, UK

[11]Healthy Lifespan Institute (HELSI), University of Sheffield, Western Bank, Sheffield, S10 2TN, UK

[12]These authors contributed equally

*Corresponding author. Email: rr360@cam.ac.uk (R.T.R), dk10012@cam.ac.uk (D.K.); S.De@sheffield.ac.uk (S.D.)

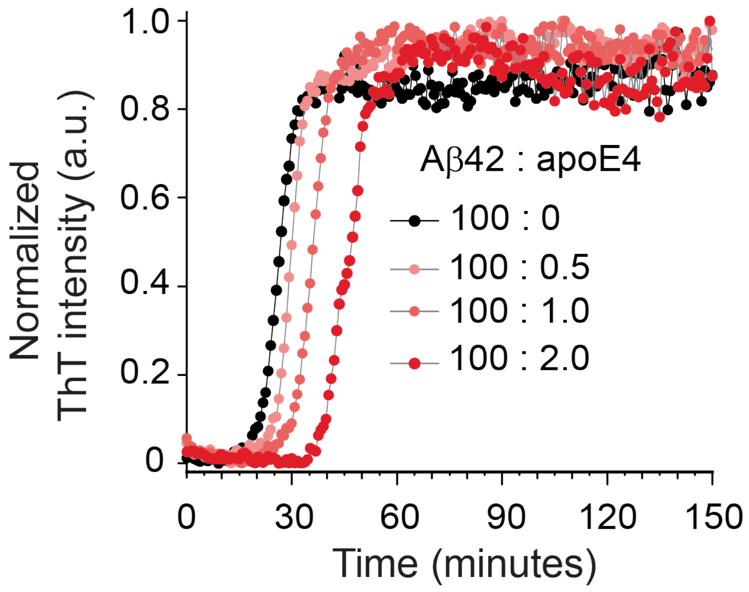

Supplementary Figure 1. Dependence of Aβ42 aggregation (4 μM) on the concentration of non-lipidated apoE4 (0, 20, 40 and 80 nM), monitored by ThT fluorescence.

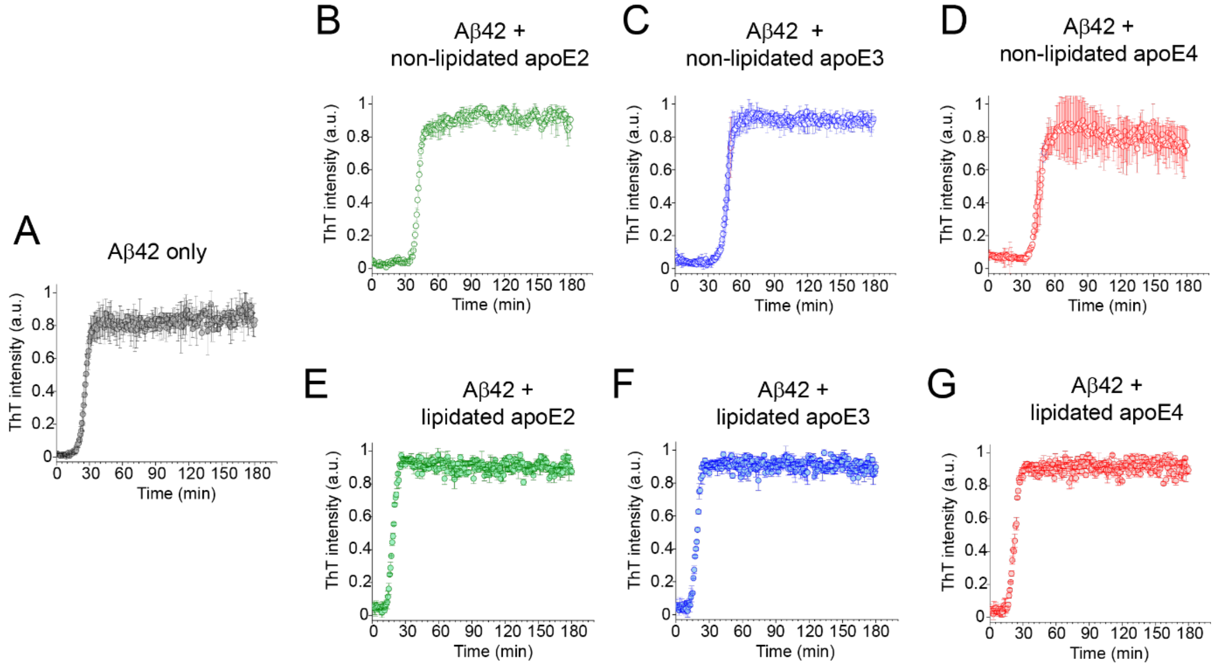

Supplementary Figure 2. Aβ42 aggregation (4 μM) in the presence of lipidated and non-lipidated isoforms of apoE (0 or 80 nM), monitored by ThT fluorescence. Data are plotted as the mean and standard deviation of three independent experiments, each using a different batch of Aβ42 peptide from the same manufacturer.

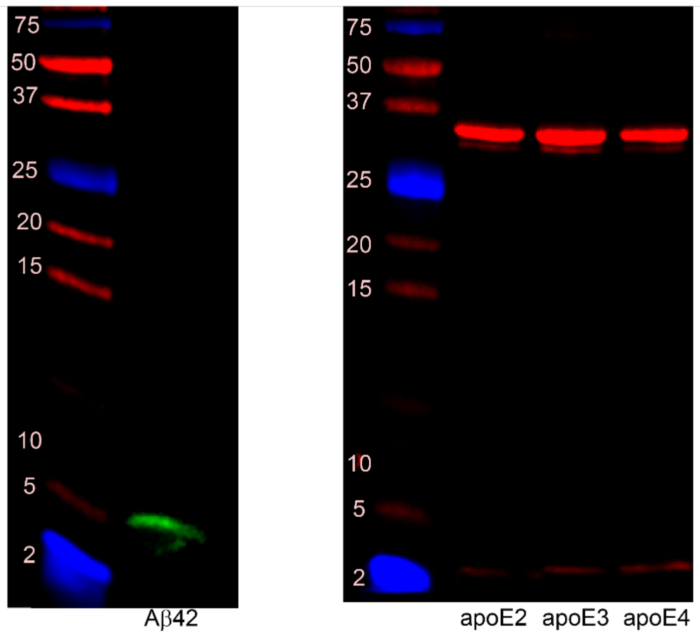

Supplementary Figure 3. Characterization of Aβ42, apoE2, apoE3 and apoE4 purity by western blotting.

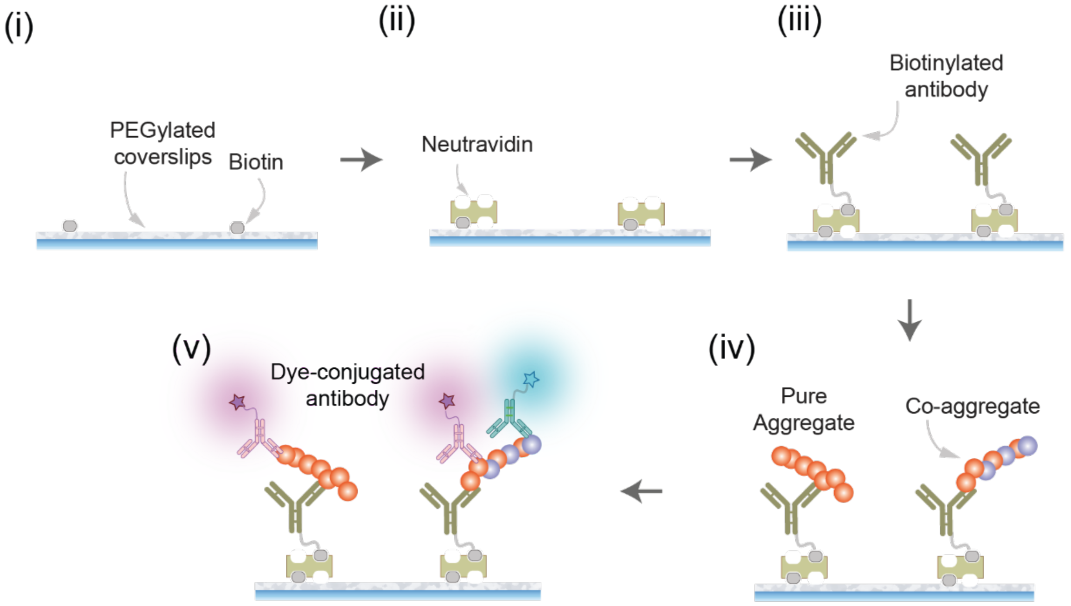

Supplementary Figure 4. Schematic of SiMPull assay.

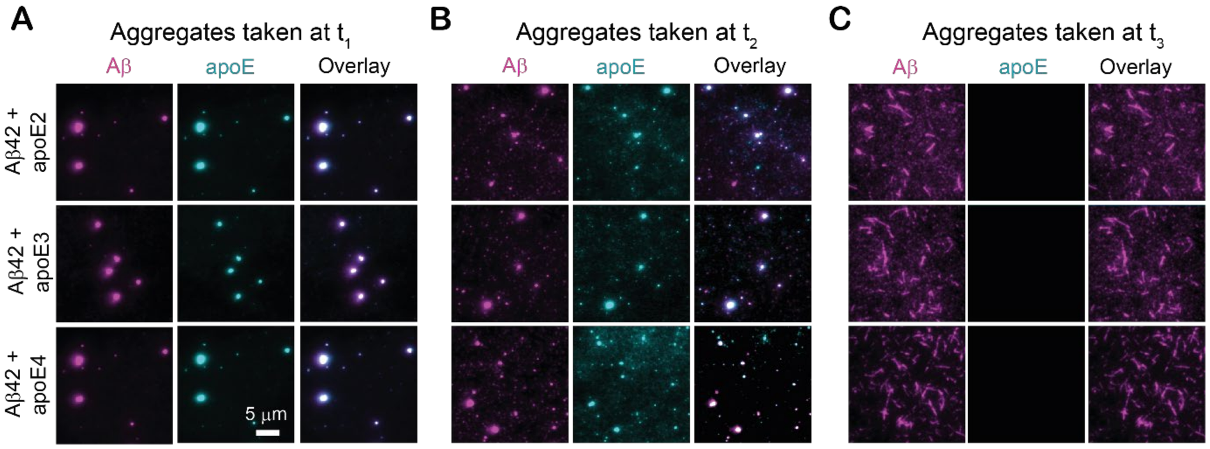

Supplementary Figure 5. Colocalization of apoE with Aβ42 aggregates does not depend on the anti-apoE antibody used. Aliquots of non-lipidated apoE-Aβ42 co-aggregation mixtures (aggregated at 4 μM Aβ42 and 80 nM non-lipidated apoE, then diluted 4-fold prior to pulldown) at t_1, t_2 and t_3 timepoints (as defined in Figure 1B) imaged using SiMPull with biotinylated 6E10 antibody for capture, and Aβ-specific Alexa-Fluor-647-labeled 6E10 (500 pM) and apoE-specific Alexa-Fluor-488-labeled F-9 (1 nM) as imaging antibodies. As in Figure 1 - where a different apoE antibody is used - apoE colocalizes with early-stage Aβ42 aggregates, but not fibrillar aggregates.

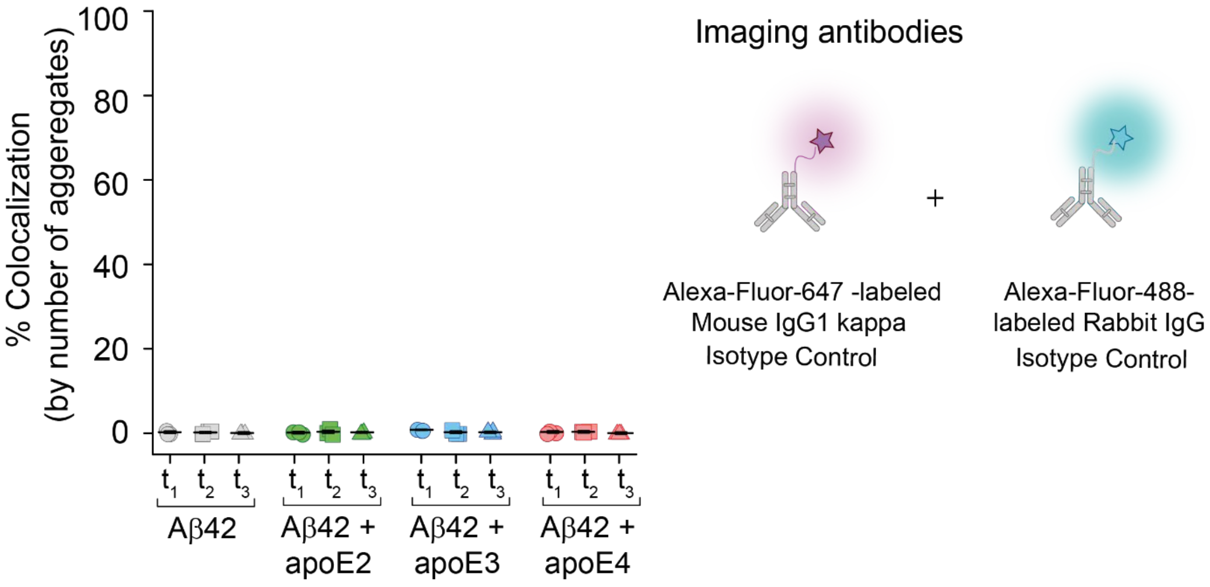

Supplementary Figure 6. Isotype control antibodies generate no colocalization in two-color SiMPull imaging. Aβ42 aggregates and non-lipidated apoE-Aβ42 co-aggregates formed at different stages of aggregation (defined in Figure 1B) and imaged with SiMPull using two isotype control antibodies. For all samples, no significant colocalization is observed.

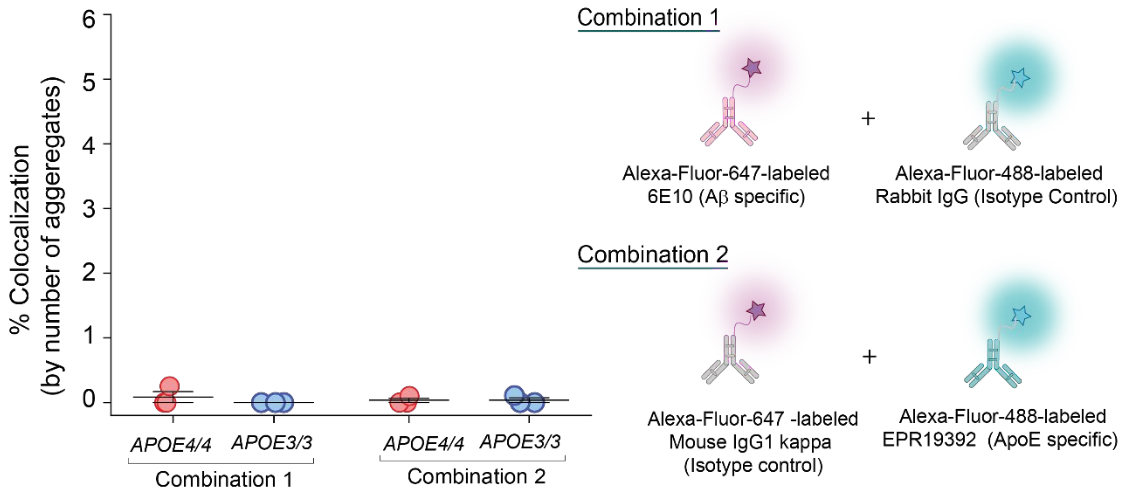

Supplementary Figure 7. Aβ and apoE antibodies bind co-aggregates *via* their specific paratopes. Aβ-containing aggregates captured from frontal cortex extracts of homozygous *APOE4* and *APOE3* AD patients imaged using two different combinations of imaging antibodies (n=3). In each experiment, one isotype control antibody is combined with an Aβ- or apoE-specific antibody respectively. No significant colocalization is observed, indicating that antibody binding is paratope mediated.

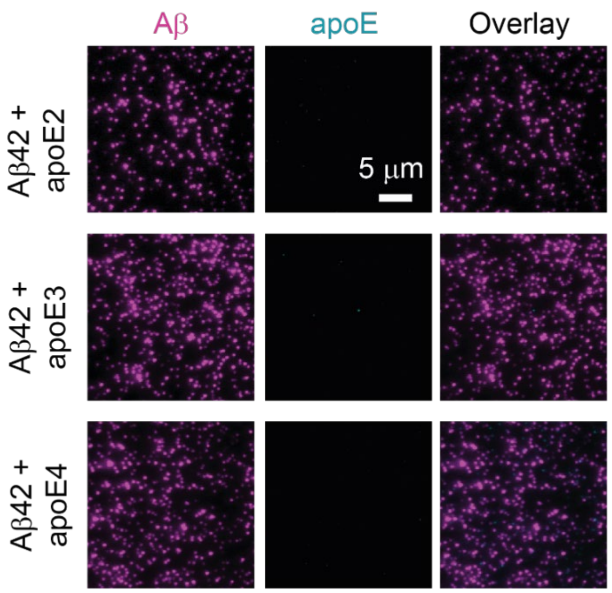

Supplementary Figure 8. ApoE does not colocalize with pre-formed Aβ42 aggregates. Aβ42 aggregates (4 μM monomer equivalents) taken at the end of the lag phase (t_1, as defined in Figure 1B), incubated in an ice bath with non-lipidated apoE (80 nM), diluted 4-fold in PBS then imaged using SiMPull.

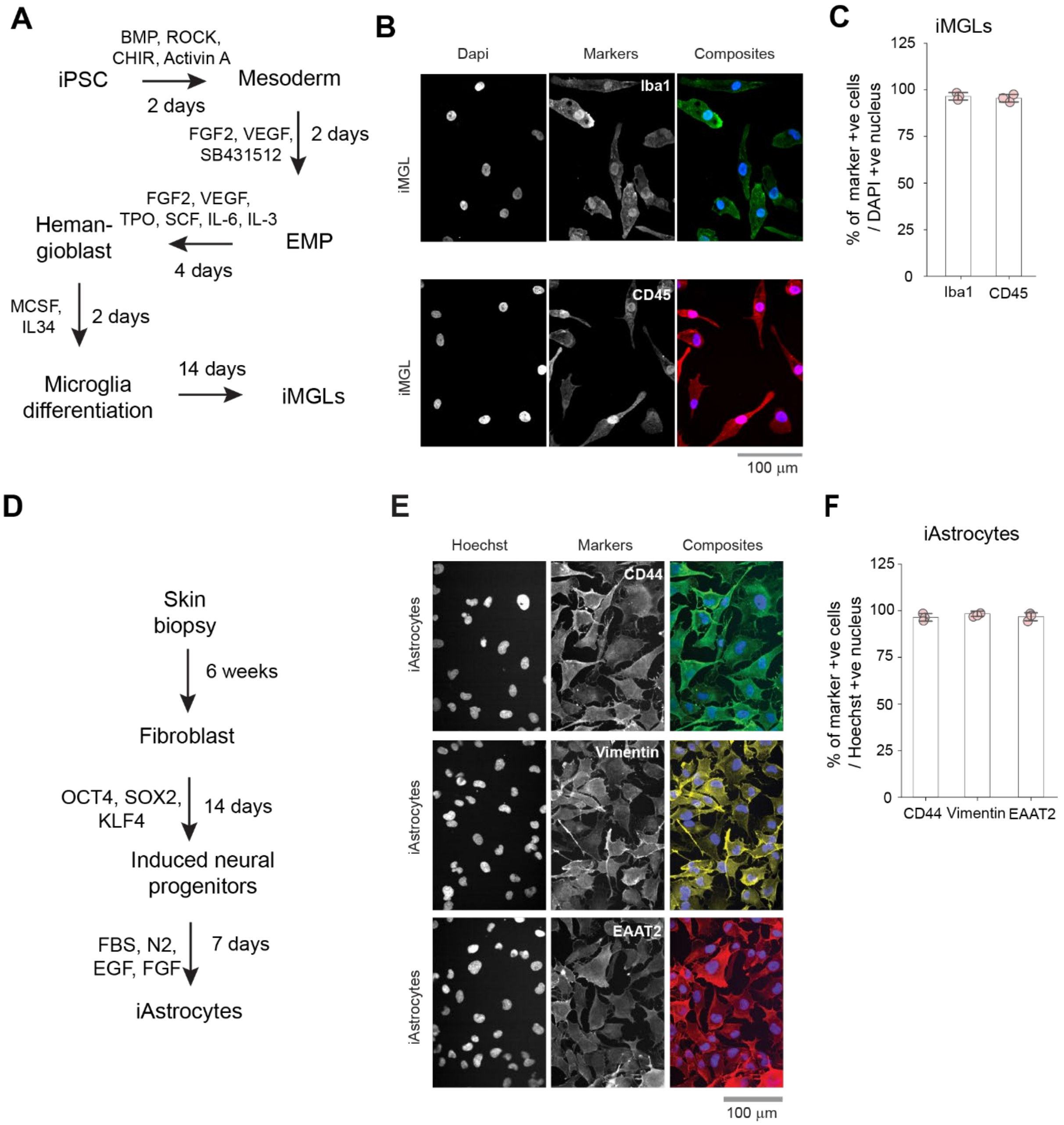

Supplementary Figure 9. Characterization of enriched populations of human-derived microglia (iMGLs) and astrocytes (iAstrocytes). (A) Differentiation protocol to generate iMGL. **(B)** Representative immunocytochemistry images showing high expression (>95%) of microglia markers Iba1 and CD45. (**C**) Quantification of immunocytochemistry images of iMGL. Data were averaged over three biological replicates; data are shown as individual data points and error bars represent standard deviation. **(D)** Differentiation protocol to generate iAstrocytes. **(E)** Representative immunocytochemistry images showing high expression (>97%) of astrocyte markers CD44, Vimentin and EAAT2. (**F**) Quantification of immunocytochemistry images of human-derived iAstrocytes. Data were averaged over three biological replicates; data are shown as individual data points and error bars represent standard deviation.

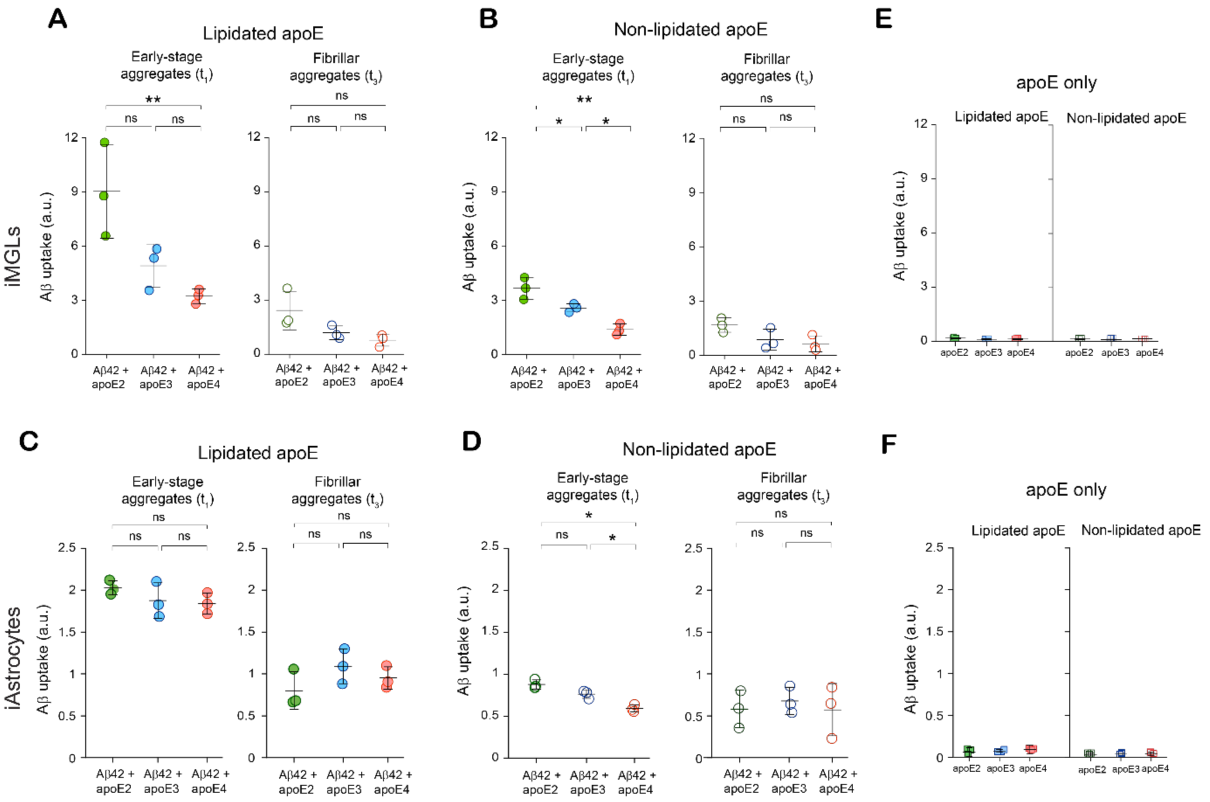

Supplementary Figure 10. Uptake of apoE-Aβ co-aggregates by human iMGLs and iAstrocytes depends on apoE isoform. Uptake of early-stage (t_1) and fibrillar (t_3) Aβ aggregates by iMGLs **(A, B)** and iAstrocytes **(C, D)** formed in the presence of lipidated **(A)** and non-lipidated apoE **(B)** and lipidated and non-lipidated apoE only by iMGLs **(E)** and iAstrocytes **(F).** Units of uptake = integrated fluorescence of sample divided by the integrated fluorescence of internalized early-stage Aβ42 aggregates at t_1 for each replicate (as in Figure 4). Data were averaged over three biological replicates for both iMGLs and iAstrocytes; data are shown as individual data points and error bars represent standard deviation. Statistical significance was calculated using a one-way ANOVA with post-hoc Tukey. $^*P < 0.05$, $^{**}P < 0.01$, $^{***}P < 0.001$, ns, non-significant ($P > 0.05$).

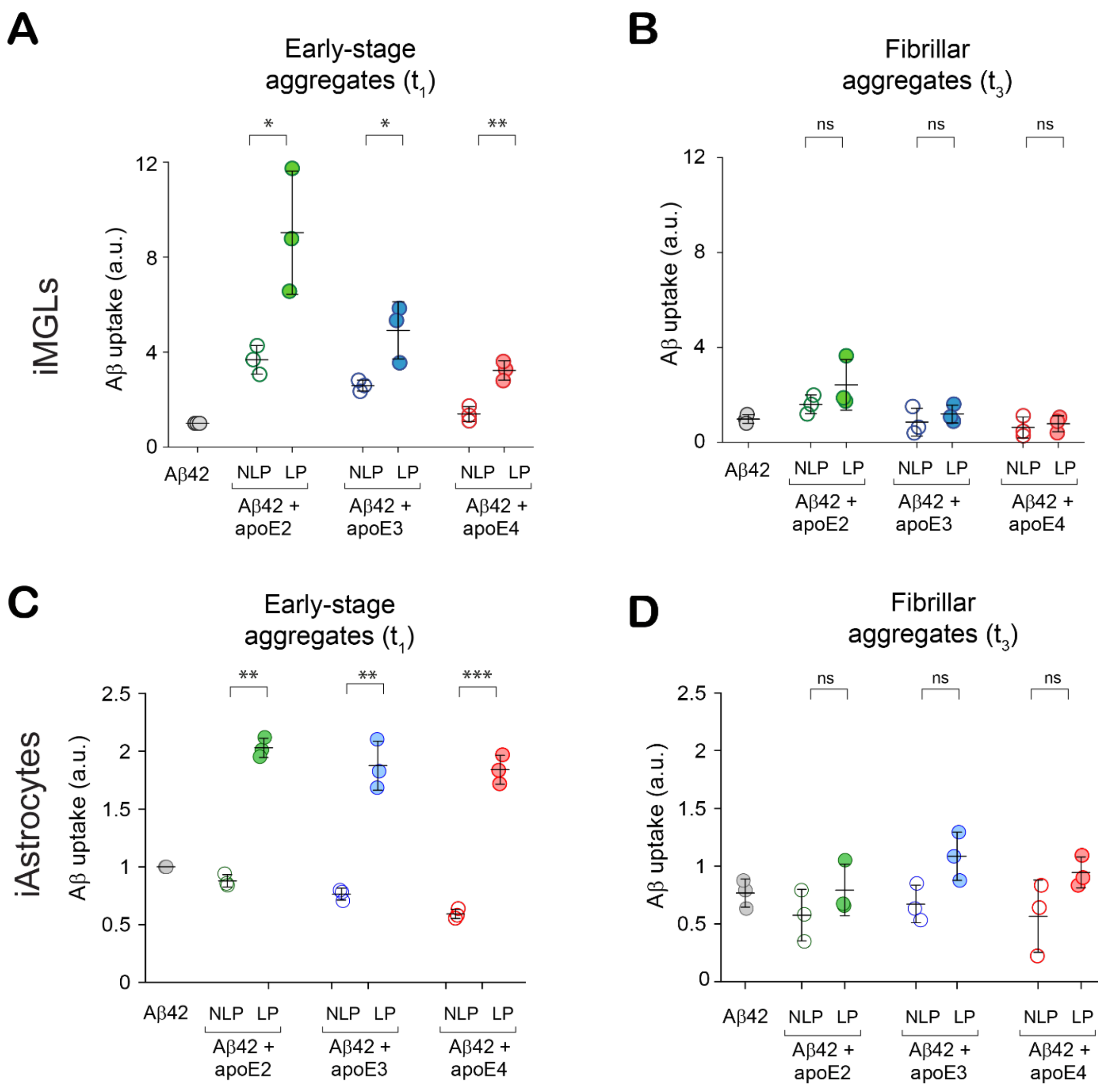

Supplementary Figure 11. Association with lipidated apoE enhances uptake of Aβ aggregates by iMGLs and iAstrocytes. Uptake of Aβ aggregates by iMGLs **(A,B)** and iAstrocytes **(C,D)** formed in the presence of lipidated and non-lipidated apoE. Units of uptake = integrated fluorescence of sample divided by the integrated fluorescence of internalized Aβ42 aggregates form at early-stage t_1 for each replicate (as in Figure 4). Data points represent one of three biological replicates; error bars represent standard deviation. Statistical significance was calculated using an unpaired two sample T-test. *$P < 0.05$, **$P < 0.01$, ***$P < 0.001$, ns, non-significant ($P > 0.05$).

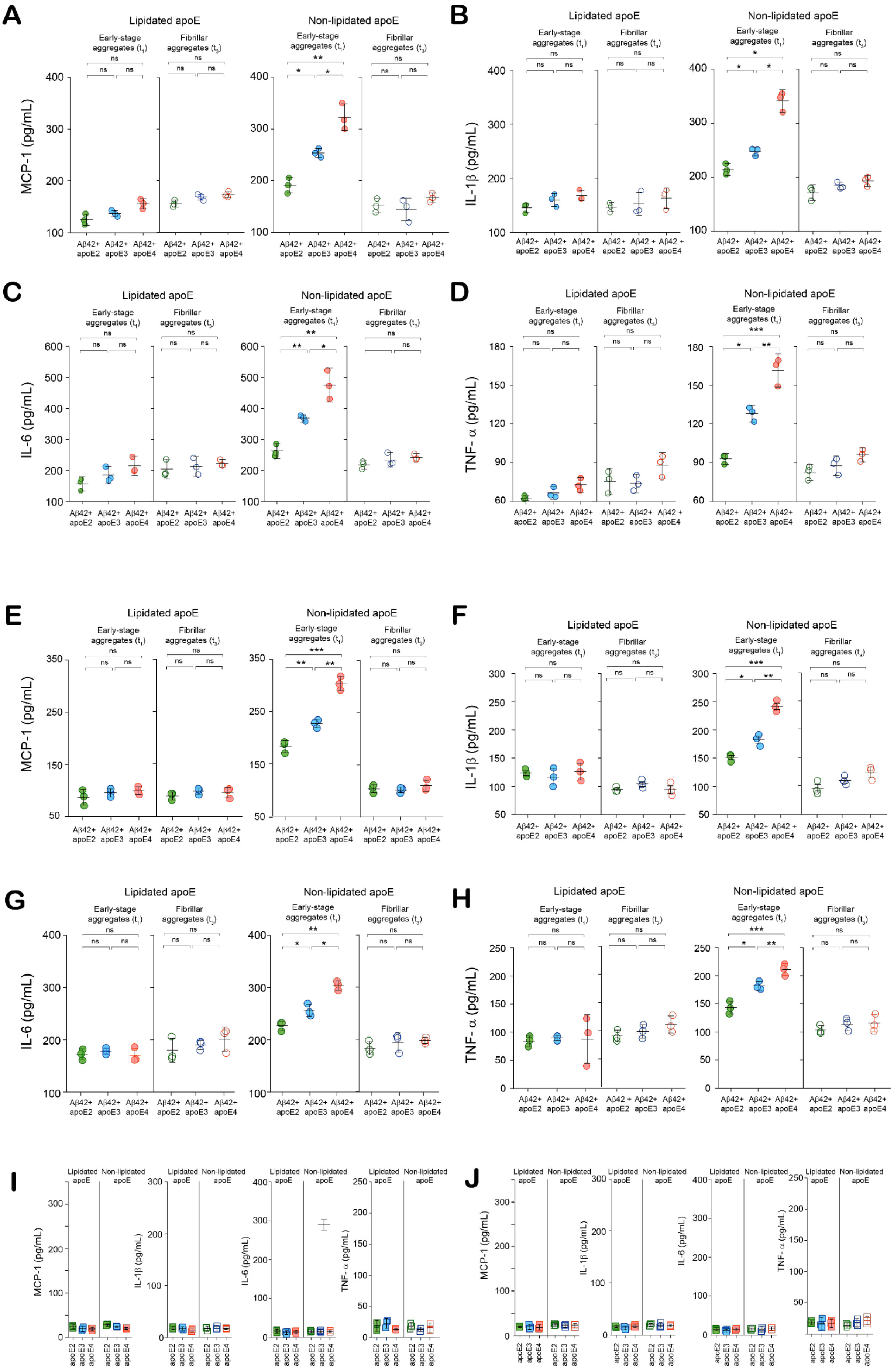

A
Lipidated apoE
Non-lipidated apoE
Early-stage aggregates (t1)
Fibrillar aggregates (t3)
MCP-1 (pg/mL)
Aβ42+ apoE2
Aβ42+ apoE3
Aβ42+ apoE4
B
IL-1β (pg/mL)
C
IL-6 (pg/mL)
D
TNF- α (pg/mL)
E
MCP-1 (pg/mL)
F
IL-1β (pg/mL)
G
IL-6 (pg/mL)
H
TNF- α (pg/mL)
I
J
Lipidated apoE
Non-lipidated apoE
apoE2
apoE3
apoE4
ns
*
**
***

Supplementary Figure 12. ApoE-Aβ co-aggregates inflame iMGLs and iAstrocytes in an isoform-dependent way. MCP-1, Il-1β, IL-6 and TNF-α release by iMGLs **(A-D)** and iAstrocytes **(E-H)** induced by early-stage (t_1), fibrillar (t_3) aggregates and lipidated and non-lipidated apoE only **(I, J)..** Each data point represents one of three biological replicates; error bars represent standard deviation. Statistical significance was calculated using one-way ANOVA with post-hoc Tukey. *P < 0.05, **P < 0.01, ***P < 0.001, ns, non-significant (P > 0.05).

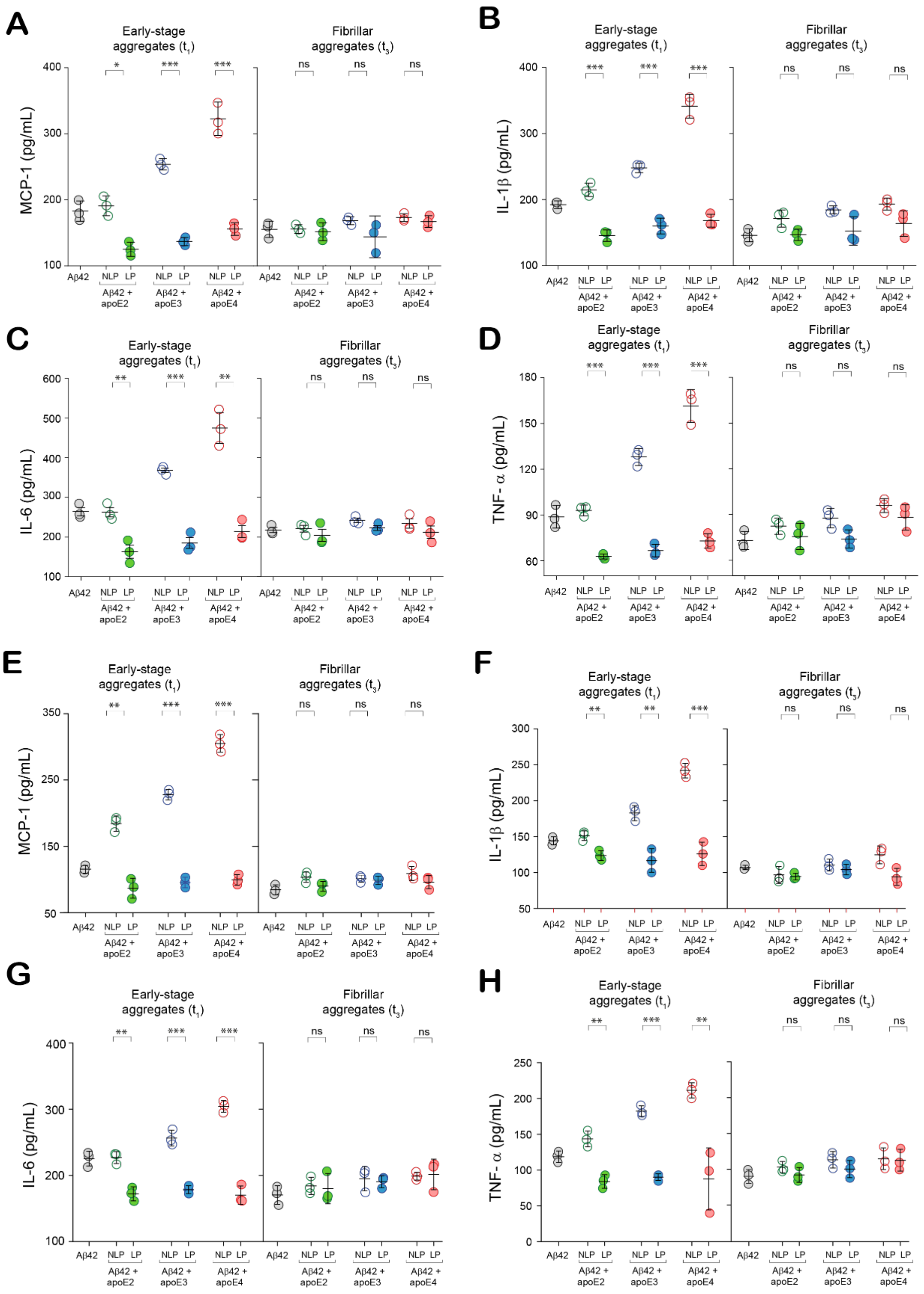

Supplementary Figure 13. ApoE-Aβ co-aggregates inflame iMGLs and iAstrocytes in an apoE-lipidation-dependent manner. MCP-1, Il-1β, IL-6 and TNF-α release by human iMGLs **(A-D)** and iAstrocytes **(E-H)** induced by early-stage (t_1) and fibrillar (t_3) aggregates. Each data point represents one of three biological replicates; error bars represent standard deviation.

Statistical significance was calculated using a two-sample t-test. *P < 0.05, **P < 0.01, ***P < 0.001, ns, non-significant (P > 0.05).

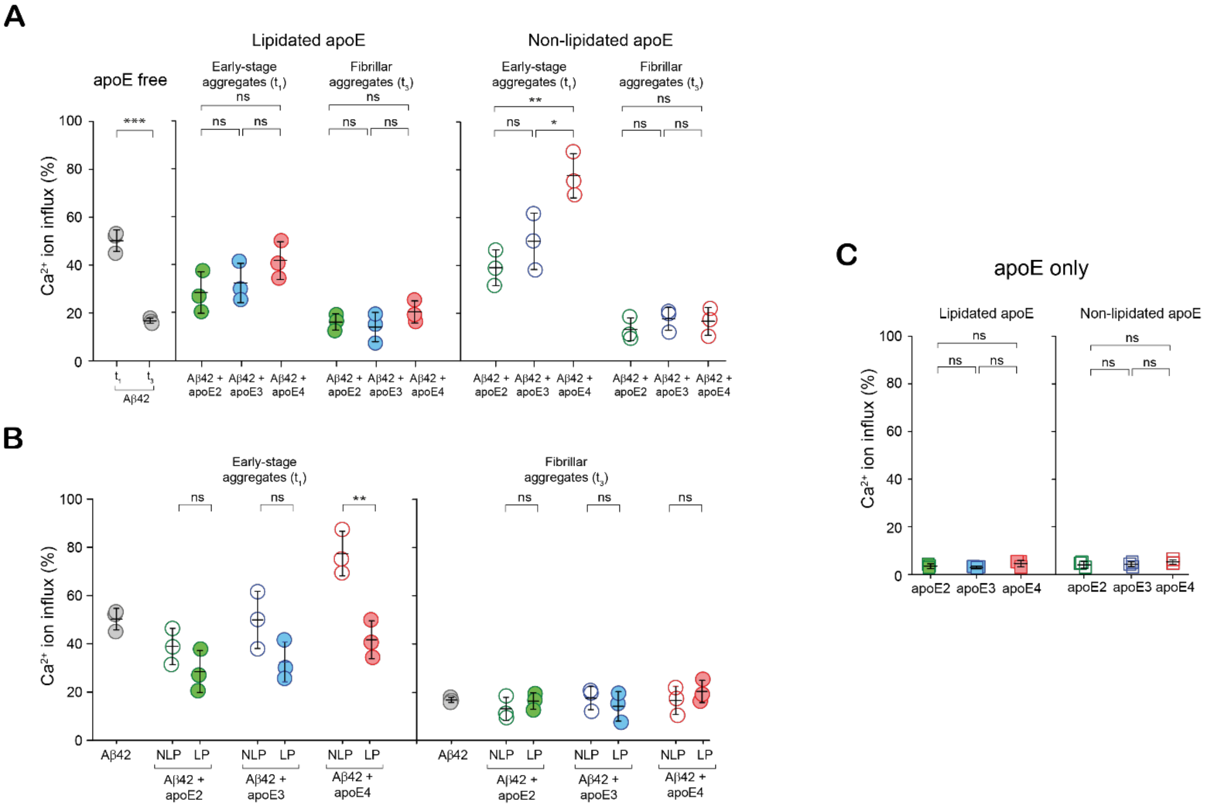

Supplementary Figure 14. Isoforms and lipidation of apoE modulate the lipid membrane permeabilization ability of Aβ aggregates differently. Comparison of lipid bilayer permeabilization by **(A)** apoE-Aβ co-aggregates containing different apoE isoforms and **(B)** lipidated and non-lipidated apoE-Aβ co-aggregates formed at t_1 (end of lag phase) and t_3 (plateau phase, where aggregates are mostly Aβ-only fibrils) ([Aβ42] = 4 μM in monomer equivalents; [apoE] = 0 or 80 nM) **(C)** Lipidated and non-lipidated apoE only. Ca^{2+} influx is referenced to the influx caused by the ionophore, ionomycin. Data points represent one of three technical replicates; error bars represent standard deviation and statistical significance was calculated using One-way ANOVA with post-hoc Tukey **(A)** and an unpaired two-sample t-test **(B)**. *P < 0.05, **P < 0.01, ***P < 0.001, NS, non-significant (P > 0.05).

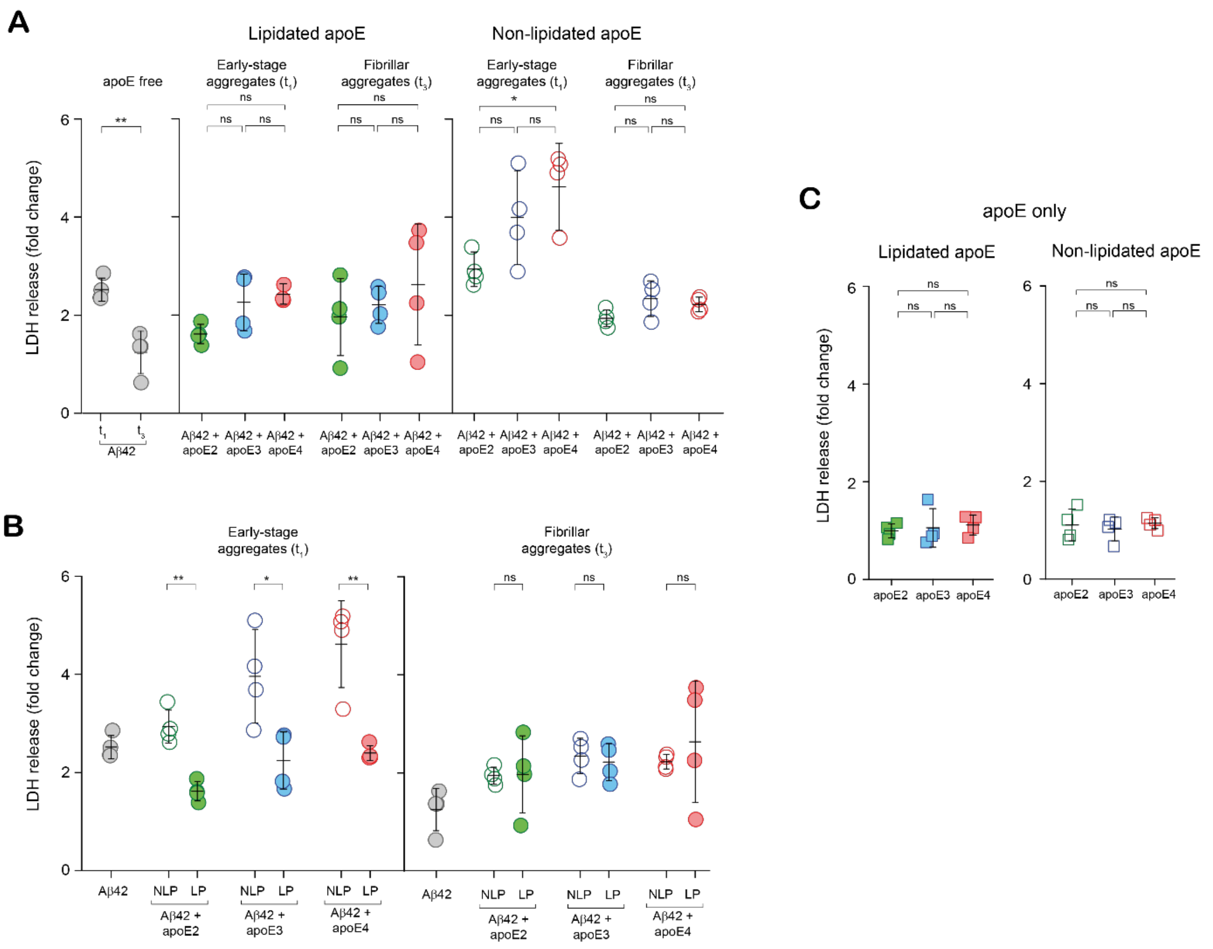

Supplementary Figure 15. Isoforms and lipidation of apoE modulate the Aβ-aggregate-induced LDH release differently. Comparison of LDH release elicited by **(A)** apoE-Aβ co-aggregates containing different apoE isoforms and **(B)** lipidated and non-lipidated apoE-Aβ co-aggregates formed at t_1 (end of lag phase) and t_3 (plateau phase, where aggregates are mostly Aβ-only fibrils) ([Aβ42] = 4 μM in monomer equivalents; [apoE] = 0 or 80 nM) **(C)** Lipidated and non-lipidated apoE only. Units of LDH release = LDH release elicited by sample divided by LDH release elicited by buffer for each replicate. Data points represent one of four biological replicates; error bars represent standard deviation and statistical significance was calculated using One-way ANOVA with post-hoc Tukey **(A)** and an unpaired two-sample t-test **(B)**. $^*P < 0.05$, $^{**}P < 0.01$, $^{***}P < 0.001$, NS, non-significant ($P > 0.05$).

Supplementary Table 1: Comparison of the aggregation kinetics of 4 μM Aβ42 measured here with previously published data.

Article	Journal	Fig. #	Lag time
Proliferation of amyloid-β42 aggregates occurs through a secondary nucleation mechanism	PNAS 2013, 110, 9758-9763	1	18 min
Direct measurement of lipid membrane disruption connects kinetics and toxicity of Aβ42 aggregation	NSMB 2020, 27,886	1b	15 min
On the role of sidechain size and charge in the aggregation of Aβ42 with familial mutations	PNAS 2018, 115, E5849-E5858	3	30 min
High-yield Production of Amyloid-β Peptide Enabled by a Customized Spider Silk Domain	Scientific Reports 2020, 235, 10	4A	24 min
Quantitative analysis of intrinsic and extrinsic factors in the aggregation mechanism of Alzheimer-associated Aβ-peptide	Scientific Reports 2016, 6, 18728	1D	26 min
Amyloid fibril formation of Arctic amyloid-β 1–42 peptide is efficiently inhibited by the BRICHOS domain	ACS Chem. Biol. 2022, 17, 2201	S3A (SI)	22 min
This work	-	-	19 ± 1.2 min

Supplementary Table 2: Numbers of particles analyzed in Figure 1.

Samples	Time point	Total number of Aβ aggregates detected	Number of Aβ-apoE co-aggregates detected	% of co-aggregates
**Aβ only**	t_1	10539	39	0.37
	t_2	4491	0	0
	t_3	2295	0	0
**Aβ + Lipidated apoE2**	t_1	2808	2084	74.21
	t_2	2268	1114	49.11
	t_3	3087	23	0.75
**Aβ + Lipidated apoE3**	t_1	4671	3677	78.72
	t_2	5058	3362	66.47
	t_3	3483	5	0.14
**Aβ + Lipidated apoE4**	t_1	5076	3667	72.24
	t_2	4473	3121	69.78
	t_3	2340	9	0.40
**Aβ + Non-lipidated apoE2**	t_1	2106	1595	75.73
	t_2	2718	862	31.71
	t_3	3611	14	0.38
**Aβ + Non-lipidated apoE3**	t_1	2114	1924	91.012
	t_2	2471	1091	44.15
	t_3	3571	21	0.58
**Aβ + Non-lipidated apoE4**	t_1	2184	1904	87.17
	t_2	2021	612	30.28
	t_3	3396	32	0.94

Supplementary Table 3: Numbers of particles analyzed in Figure 3.

Patient	Total number of Aβ aggregates detected	Number of Aβ-apoE co-aggregates detected	% of co-aggregates
APOE4/4 #1	6359	318	5.00
APOE4/4 #2	5252	196	3.72
APOE4/4 #3	6219	302	4.86
APOE3/3 #1	1671	28	1.66
APOE3/3 #2	3450	31	0.90
APOE3/3 #3	3449	26	0.76

Supplementary Table 4: the statistical significance of the observed differences in cytokine and chemokine secretion by iMGLs and iAstrocytes upon treatment with lipidated and non-lipidated apoE, determined using an unpaired two-sample t-test. (ns P > 0.05; * P ≤ 0.05; ** P ≤ 0.01; *** P ≤ 0.001; **** P ≤ 0.0001)

**Lipidated vs non-lipidated apoE**						
			t_1		t_3	
**iMGLs**	apoE2	MCP-1	**	0.00348	ns	0.65618
		IL-1β	***	8.48472E-4	ns	0.05116
		IL6	**	0.00807	ns	0.39749
		TNF-α	***	1.73548E-4	ns	0.3189
	apoE3	MCP-1	****	4.33946E-5	ns	0.13767
		IL-1β	***	3.98579E-4	ns	0.06949
		IL6	***	2.47292E-4	ns	0.08063
		TNF-α	***	1.04329E-4	ns	0.05351
	apoE4	MCP-1	***	4.35846E-4	ns	0.38249
		IL-1β	***	1.2563E-4	ns	0.07419
		IL6	**	0.00102	ns	0.32545
		TNF-α	***	2.04068E-4	ns	0.22724

**Lipidated vs nonlipidated apoE**						
			t_1		t_3	
**iAstrocytes**	**apoE2**	MCP-1	***	8.66559E-4	ns	0.09383
		IL-1β	**	0.00754	ns	0.79031
		IL6	**	0.00236	ns	0.79928
		TNF-α	**	0.00219	ns	0.22349
	**apoE3**	MCP-1	****	<0.0001	ns	0.64841
		IL-1β	**	0.00414	ns	0.3882
		IL6	***	5.28697E-4	ns	0.70244
		TNF-α	***	<0.0001	ns	0.25001
	**apoE4**	MCP-1	****	<0.0001	ns	0.18977
		IL-1β	***	4.70655E-4	ns	0.07683
		IL6	***	1.45585E-4	ns	0.85831
		TNF-α	**	0.00858	ns	0.88185

Supplementary Table 5: Statistical significance of the differences in the uptake of Aβ by iMGLs and iAstrocytes, distinguishing among different apoE isoforms (ns P > 0.05; * P ≤ 0.05; ** P ≤ 0.01; *** P ≤ 0.001; **** P ≤ 0.0001)

		iMGLs				iAstrocytes			
		Lipidated		Non-lipidated		Lipidated		Non-lipidated	
t_1	**apoE2 vs apoE3**	0.05306	ns	0.04278	*	0.46244	ns	0.02545	*
	**apoE2 vs apoE4**	0.01261	*	0.00129	**	0.33409	ns	9.37728E-4	***
	**apoE3 vs apoE4**	0.47588	ns	0.02902	*	0.95571	ns	0.01238	*
t_3	**apoE2 vs apoE3**	0.14705	ns	0.21897	ns	0.23036	ns	0.88049	ns
	**apoE2 vs apoE4**	0.05773	ns	0.1063	ns	0.62586	ns	0.99885	ns
	**apoE3 vs apoE4**	0.75137	ns	0.84098	ns	0.66082	ns	0.85925	ns

Supplementary Table 6: Statistical significance of the differences in cytokine and chemokine secretion by iMGLs and iAstrocytes, categorized by various apoE isoforms. These differences were evaluated using a one-way ANOVA, followed by a post-hoc Tukey analysis for mean comparisons (ns P > 0.05; * P ≤ 0.05; ** P ≤ 0.01; *** P ≤ 0.001; **** P ≤ 0.0001).

			iMGLs				iAstrocytes			
			**Lipidated**		**Non-lipidated**		**Lipidated**		**Non-lipidated**	
t_1	**apoE2 vs apoE3**	MCP-1	0.31962	ns	0.01145	*	0.61472	ns	0.0071	**
		IL-1β	0.26489	ns	0.04108	*	0.818	ns	0.01437	*
		IL6	0.35322	ns	0.01113	*	0.7471	ns	0.02626	*
		TNF-α	0.4588	ns	0.00261	**	0.95477	ns	0.00731	**
	**apoE2 vs apoE4**	MCP-1	0.01417	ns	2.40E-4	***	0.38458	ns	<0.0001	****
		IL-1β	0.07751	ns	4.39E-5	****	0.9735	ns	<0.0001	****
		IL6	0.05083	ns	2.92E-4	***	0.97516	ns	1.9873E-4	***
		TNF-α	0.03598	ns	6.49E-5	****	0.98481	ns	5.82585E-4	***
	**apoE3 vs apoE4**	MCP-1	0.09447	ns	0.00741	**	0.89553	ns	3.67117E-4	***
		IL-1β	0.61958	ns	2.52E-4	***	0.69667	ns	3.86026E-4	***
		IL6	0.33545	ns	0.0105	*	0.62608	ns	0.00272	**
		TNF-α	0.1774	ns	0.00333	**	0.99157	ns	0.02649	*
t_3	**apoE2 vs apoE3**	MCP-1	0.83008	ns	0.10756	ns	0.41041	ns	0.93359	ns
		IL-1β	0.91258	ns	0.29986	ns	0.39611	ns	0.33413	ns
		IL6	0.90391	ns	0.4226	ns	0.80365	ns	0.60532	ns

		TNF-α	0.96381	ns	0.50828	ns	0.72556	ns	0.57339	ns
	**apoE2 vs apoE4**	MCP-1	0.49168	ns	0.03219	ns	0.63992	ns	0.69256	ns
		IL-1β	0.5007	ns	0.07485	ns	0.99661	ns	0.04679	ns
		IL6	0.61065	ns	0.19106	ns	0.42132	ns	0.42117	ns
		TNF-α	0.19793	ns	0.05357	ns	0.19934	ns	0.49716	ns
	**apoE3 vs apoE4**	MCP-1	0.24843	ns	0.61861	ns	0.90103	ns	0.49654	ns
		IL-1β	0.72842	ns	0.54776	ns	0.36207	ns	0.32758	ns
		IL6	0.84764	ns	0.79388	ns	0.76749	ns	0.93396	ns
		TNF-α	0.14239	ns	0.23661	ns	0.50064	ns	0.9889	ns

Supplementary Table 7: Statistical relevance of difference in membrane damage caused by Aβ aggregated forms, both in the presence and absence of various lipidation states and isoforms of apoE. The one-way ANOVA, succeeded by a post-hoc Tukey analysis, was employed for the purpose of mean comparisons. (ns P > 0.05; * P ≤ 0.05; ** P ≤ 0.01; *** P ≤ 0.001; **** P ≤ 0.0001).

Isoform dependence						
	Non-lipidated apoE			Lipidated apoE		
**t_1**	**apoE2 vs apoE3**	0.06016	ns	**apoE2 vs apoE3**	0.83065	ns
	**apoE2 vs apoE4**	0.00667	**	**apoE2 vs apoE4**	0.19873	ns
	**apoE3 vs apoE4**	0.03057	*	**apoE3 vs apoE4**	0.40445	ns
**t_3**	**apoE2 vs apoE3**	0.57389	ns	**apoE2 vs apoE3**	0.85715	ns
	**apoE2 vs apoE4**	0.70233	ns	**apoE2 vs apoE4**	0.5729	ns
	**apoE3 vs apoE4**	0.97192	ns	**apoE3 vs apoE4**	0.3194	ns
