# Supplementary material for: Co-aggregation with Apolipoprotein E modulates the function of Amyloid-β in Alzheimer’s disease
Source: Nat Commun. 2024 Jun 1;15:4695. doi: 10.1038/s41467-024-49028-z (PMC11144216; doi:10.1038/s41467-024-49028-z)
Supplement: Supplementary file 3 — Reporting Summary [file 41467_2024_49028_MOESM3_ESM.pdf]

Corresponding author(s): Rohan T Ranasinghe, David Klenerman, Suman De

Last updated by author(s): Apr 11, 2024

Reporting Summary

Nature Portfolio wishes to improve the reproducibility of the work that we publish. This form provides structure for consistency and transparency in reporting. For further information on Nature Portfolio policies, see our Editorial Policies and the Editorial Policy Checklist.

Statistics

For all statistical analyses, confirm that the following items are present in the figure legend, table legend, main text, or Methods section.

n/a	Confirmed	
☐	☒	The exact sample size (n) for each experimental group/condition, given as a discrete number and unit of measurement
☐	☒	A statement on whether measurements were taken from distinct samples or whether the same sample was measured repeatedly
☐	☒	The statistical test(s) used AND whether they are one- or two-sided<br>*Only common tests should be described solely by name; describe more complex techniques in the Methods section.*
☐	☒	A description of all covariates tested
☒	☐	A description of any assumptions or corrections, such as tests of normality and adjustment for multiple comparisons
☐	☒	A full description of the statistical parameters including central tendency (e.g. means) or other basic estimates (e.g. regression coefficient) AND variation (e.g. standard deviation) or associated estimates of uncertainty (e.g. confidence intervals)
☐	☒	For null hypothesis testing, the test statistic (e.g. F, t, r) with confidence intervals, effect sizes, degrees of freedom and P value noted<br>*Give P values as exact values whenever suitable.*
☒	☐	For Bayesian analysis, information on the choice of priors and Markov chain Monte Carlo settings
☒	☐	For hierarchical and complex designs, identification of the appropriate level for tests and full reporting of outcomes
☒	☐	Estimates of effect sizes (e.g. Cohen's d, Pearson's r), indicating how they were calculated

Our web collection on statistics for biologists contains articles on many of the points above.

Software and code

Policy information about availability of computer code

Data collection	Acquisition of single aggregate imaging data were performed using Micro-manager 2.0, Cell imaging data acquired using High content PerkinElmer Opera Phenix software. Protein-aggregation, LDH cytotoxicty and ELISA data were collected using BMG-labtech Plate-reader software. Details are provided in the methods section.
Data analysis	Single aggregate imaging data analysis were performed using a custom made script written in python based on Comdet plugin of Fiji. Cell imaging data were analyzed using PerkinElmer Harmony Analysis software. Data analysis and plotting were performed using Origin 9.0 and finally figures are edited using Adobe Illustrator CS6.

For manuscripts utilizing custom algorithms or software that are central to the research but not yet described in published literature, software must be made available to editors and reviewers. We strongly encourage code deposition in a community repository (e.g. GitHub). See the Nature Portfolio guidelines for submitting code & software for further information.

Data

Policy information about availability of data

All manuscripts must include a data availability statement. This statement should provide the following information, where applicable:

- Accession codes, unique identifiers, or web links for publicly available datasets
- A description of any restrictions on data availability
- For clinical datasets or third party data, please ensure that the statement adheres to our policy

All data made publicly available upon publication. All the analysed data were deposited in a spreadsheet with the manuscript
Custom scripts used to run the colocalization analyses described in this study are available via Github.
https://github.com/zengjiexia/CoincidenceAnalysis
https://github.com/zengjiexia/CellIntakeAnalysis

All data are available from the corresponding authors upon request. Source data are provided with this paper.

Human research participants

Policy information about studies involving human research participants and Sex and Gender in Research.

Reporting on sex and gender	Sex information for all patients and control can be found on supplementary table 3.
Population characteristics	Brain tissue collection procedures from the control and patients were approved by the Ihe Newcastle and North Tyneside Research Ethics Committee UK, and written consent for brain donation was obtained from patients or their surrogate decision makers.
Recruitment	Human tissue samples were not collected de novo during this project. Postmortem tissues were provided by the Newcastle Brain Tissue Resource, UK under Material Transfer Agreement. The clinical data related to the tissue supplied were made available on an anonymized basis. For Alzheimer's disease brain tissue, a histological diagnostic postmortem evaluation was performed to confirm the presence of Alzheimer's disease
Ethics oversight	The ethical approval were granted through the Newcastle and North Tyneside Research Ethics Committee.

Note that full information on the approval of the study protocol must also be provided in the manuscript.

Field-specific reporting

Please select the one below that is the best fit for your research. If you are not sure, read the appropriate sections before making your selection.

☒ Life sciences ☐ Behavioural & social sciences ☐ Ecological, evolutionary & environmental sciences

For a reference copy of the document with all sections, see nature.com/documents/nr-reporting-summary-flat.pdf

Life sciences study design

All studies must disclose on these points even when the disclosure is negative.

Sample size	No statistical methods were used to pre-determine sample sizes
Data exclusions	No data were excluded from the analysis. The results of all the replicates are consistently similar to each other.
Replication	All the experiments were done with minimum of 3 biological replicates. Numbers of replicates are listed in each figure captions.
Randomization	All the protein-aggregate and cell imaging data were acquired using automatic microscope stage movement to avoid any user bias. For other experiments, this is not relevant.
Blinding	Blinding was not relevant to our study.

Reporting for specific materials, systems and methods

We require information from authors about some types of materials, experimental systems and methods used in many studies. Here, indicate whether each material, system or method listed is relevant to your study. If you are not sure if a list item applies to your research, read the appropriate section before selecting a response.

Materials & experimental systems

n/a	Involved in the study
☐	☒ Antibodies
☐	☒ Eukaryotic cell lines
☒	☐ Palaeontology and archaeology
☒	☐ Animals and other organisms
☒	☐ Clinical data
☒	☐ Dual use research of concern

Methods

n/a	Involved in the study
☒	☐ ChIP-seq
☒	☐ Flow cytometry
☒	☐ MRI-based neuroimaging

Antibodies

Antibodies used

Following antibodies were used without further modification or purification.

Biotinylated 6E10 (Biolegend, Cat. No. 803007, Lot No. B230416)
Alexa-Fluor-647-labeled 6E10 (Biolegend, Cat. No. 803021, Lot No. B304121)
Alexa-Fluor-488-labeled anti-apoE antibody F-9 (Santa Cruz Biology, Cat. No. sc-390925448 AF488, Lot No. B1717)
Alexa-Fluor-647-labeled Mouse IgG1 (isotype control, MGL, Cat. No. M075-A64, Lot. No. 002)
Alexa-Fluor-488-labeled Mouse IgG (isotype control, Fisher Scientific, Cat. No. 65-0865-14)

Anti-apoE antibody EPR19392 (Abcam, Cat. No. ab227993, Lot No. GR3268327-5,1) was labeled with Alexa Fluor 488 TFP ester and used for imaging.

Validation

All the antibodies used in this work are validated by multiple groups all over the world

Eukaryotic cell lines

Policy information about cell lines and Sex and Gender in Research

Cell line source(s)

iAstrocytes: Skin fibroblasts were collected by the Sheffield biobank, UK from individuals and Professor Laura Ferraruilo's lab convert it to iNPC. Then we reprogmmed this cell to iAstrocytes.
iMicroglia: Fibroblasts were provided by Professor Tarja Malm's lab and we have reprogrammed to iMicroglia in our lab.

Authentication

iMicroglia and iAstrocytes were validated by immunocytochemistry with well validated markers and also previously used for peer-reviewed publications.

Mycoplasma contamination

All lines were regularly tested and confirmed to be negative for mycoplasma using the Mycoplasma Detection Kit

Commonly misidentified lines
(See ICLAC register)

No commonly misidentified lines were used for this study.
